# Synthesis of Hybrid Polyphenol/Hydroxyapatite Nanomaterials with Anti-Radical Properties

**DOI:** 10.3390/nano12203588

**Published:** 2022-10-13

**Authors:** Estelle Palierse, Sylvie Masse, Guillaume Laurent, Patrick Le Griel, Gervaise Mosser, Thibaud Coradin, Claude Jolivalt

**Affiliations:** 1 Sorbonne Université, CNRS, Laboratoire de Chimie de la Matière Condensée de Paris, 75005 Paris, France; 2 Sorbonne Université, CNRS, Laboratoire de Réactivité de Surface, 75005 Paris, France

**Keywords:** hydroxyapatite, polyphenol, crystal growth

## Abstract

Plant-derived natural bioactive molecules are of great therapeutic potential but, so far, their application in nanomedicine has scarcely been studied. This work aimed at comparing two methodologies, i.e., adsorption and in situ incorporation, to prepare hybrid polyphenol/hydroxyapatite nanoparticles. Two flavonoids, baicalin and its aglycone derivative baicalein, and two phenolic acids derived from caffeic acid, rosmarinic and chlorogenic acids, were studied. Adsorption of these polyphenols on pre-formed hydroxyapatite nanoparticles did not modify particle size or shape and loading was less than 10% (*w*/*w*). In contrast, presence of polyphenols during the synthesis of nanoparticles significantly impacted and sometimes fully inhibited hydroxyapatite formation but recovered particles could exhibit higher loadings. For most hybrid particles, release profiles consisted of a 24 h burst effect followed by a slow release over 2 weeks. Antioxidant properties of the polyphenols were preserved after adsorption but not when incorporated in situ. These results provide fruitful clues for the valorization of natural bioactive molecules in nanomedicine.

## 1. Introduction

**Figure 1:**
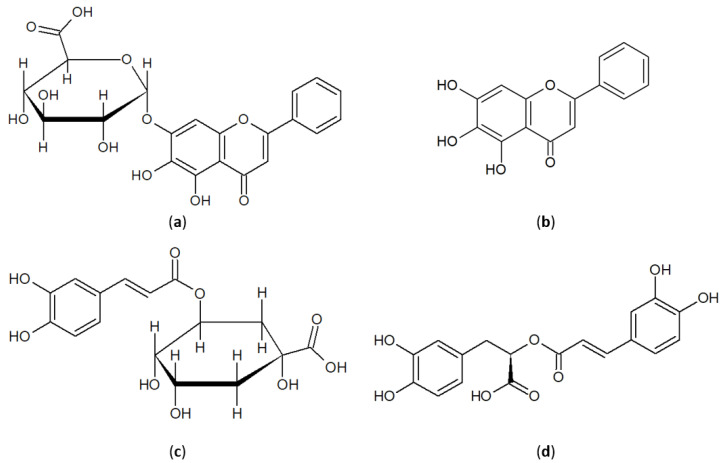
Molecular structures of polyphenols. (**a**) Baicalin—BA; (**b**) baicalein—E; (**c**) chlorogenic acid—CA; (**d**) rosmarinic acid—RA.

After many years of the use of synthetic or hemi-synthetic molecules in the pharmaceutical area, attention has recently turned towards natural molecules, mainly those extracted from plants, that exhibit many advantages. For example, polyphenols are secondary metabolites widespread in plants where they play a role of defense against pathogens [1,2]. They can be separated in two groups: flavonoids and non-flavonoids. Flavonoids are composed of a carbon skeleton of two benzene rings, A and B, linked by a linear three-carbon chain that forms a closed pyran ring (ring C) with benzene ring A. Among them, baicalin (BA) and its aglycon derivative baicalein (BE) (Figure 1a,b) are extracted from the root of the plant *Scutellaria baicalensis* and are used in traditional Chinese medicine. BA and BE have been shown to exhibit anti-inflammatory, antioxidant, antimicrobial, and anticancer properties [3,4,5,6,7,8]. Non-flavonoid polyphenols are phenolic acids, such as two derivatives of caffeic acid, rosmarinic acid (RA) and chlorogenic acid (CA) (Figure 1c,d). CA is an ester of caffeic acid with (L)-quinic acid and is found in many foods and beverages, mainly coffee, fruits, vegetables, and lamiacae (such as oregano and thyme) [9,10]. It is known for its antioxidant and antibacterial properties [11,12,13]. Rosmarinic acid is an ester of caffeic acid with 3-(3,4-dihydroxyphenyl) lactic acid found in many culinary herbs such as rosemary, with anticancer, antimicrobial, and antioxidant properties [14,15,16].

Hydroxyapatite nanoparticles (HAp NPs) are being developed for a wide range of medical applications [17,18], especially for bone regeneration [19,20]. They are often associated with bioactive compounds, such as antioxidants [21,22], osteogenic factors [23,24,25], anti-inflammatory drugs [26,27], antimicrobial agents [28,29], or antifibrinolytics [24]. The two main methods to combine bioactive molecules and HAp NPs are adsorption on pre-formed particles or incorporation during their synthesis. In the case of adsorption, the loading rate is controlled by the interactions between the active component and the hydroxyapatite surface [30,31,32,33]. For example, it is well-known that molecules, such as bisphosphonates, have a strong affinity for hydroxyapatite surfaces [23,24,25]. Other functional groups, such as carboxylic acids or phenols, have also been shown to bind hydroxyapatite surfaces [34,35], allowing for the efficient adsorption of natural and synthetic antibiotics or anti-inflammatory molecules [26,36]. Synthesis of HAp in the presence of polyphenols, such as gallic acid or tannic acid [37,38], has also been reported but led to a partial or complete inhibition of hydroxyapatite crystallization, which was attributed either to complexation of Ca^2+^ or adsorption on the surface of hydroxyapatite seeds. However, to our knowledge, these two approaches have never been compared using the same polyphenols at comparable concentrations and, in particular, their relative benefits in terms of hydroxyapatite formation and molecule loading have not yet been studied. Here, we compare these two approaches (adsorption and in situ) for the incorporation of four polyphenols, BA, BE, CA, and RA, on/in HAp nanoparticles. Besides their biological activity that may contribute to bone remodeling [39], these four molecules possess carboxylic acid or catechol functions that are known to bind the hydroxyapatite surface or complex the Ca^2+^ ion. For these reasons, they are candidates of choice to be incorporated in hydroxyapatite nanoparticles with the aim to be used in nanomedicine where their intrinsic properties may complement the osteoconductive properties of HAp.

## 2. Materials and Methods

### 2.1. Chemicals

Chlorogenic acid (CA) and rosmarinic acid (RA) was purchased from Carbosynth Limited (Compton, Berkshire, UK). Baicalein (BE) and baicalin (BA) were purchased from TCI Europe (Zwijndrecht, Belgium). Stock solutions of the four molecules (0.1 M) were prepared in dimethylsulfoxide (DMSO, Merck KGaA, Darmstadt, Germany).

### 2.2. Hydroxyapatite Synthesis

Pure hydroxyapatite was synthesized by co-precipitation from calcium hydroxide and ammonium dihydrogenophosphate (Merck KGaA, Darmstadt, Germany) solutions [34]. Briefly, Ca(OH)_2_ (7.4 g) was suspended in 100 mL of distilled water over 1.5 h. Then, 50 mL of an aqueous solution of NH_4_H_2_PO_4_ (1.2 M) was slowly added to the calcium hydroxide solution and the mixture was left to react for 48 h at 25 °C while stirring. The resulting particles were recovered by centrifugation at 5000 rpm for 5 min, washed three times with distilled water, and dried at 100 °C for 24 h.

### 2.3. Hybrid Particle Synthesis

#### 2.3.1. Adsorption Method

For adsorption experiments, the polyphenol concentration was set to 0.05 mM, a value close to the apparent solubility of BA and BE (ca. 0.1 mM) in distilled water. The hydroxyapatite particle weight was then calculated to obtain a polyphenol-to-Ca molar ratio of 1:1 in the reaction mixture. On this basis, 10 mg of HAp NPs were suspended in 200 mL of the 0.5 mM polyphenol solution and stirred at room temperature for 24 h. Particles were then recovered by centrifugation and dried at 100 °C for 24 h.

#### 2.3.2. In Situ Incorporation

In these experiments, two polyphenol concentrations, 0.05 mM and 0.5 mM, were tested. To avoid too small polyphenol-to-Ca ratios, initial concentrations of Ca(OH)_2_ and NH_4_H_2_PO_4_ were decreased by a factor of 100 compared to the above-described protocol. On this basis, polyphenols were dissolved in 100 mL of a 10 mM calcium hydroxide solution for 1.5 h before addition of 50 mL of a 12 mM ammonium dihydrogenophosphate solution. After stirring at room temperature for 24 h, particles were recovered by centrifugation and dried at 100 °C for 24 h.

### 2.4. Characterization Methods

#### 2.4.1. Thermogravimetric Analysis (TGA)

Thermogravimetric analysis (TGA) was performed with an SDT Q600 apparatus from TA Instruments (Newcastle, DE, USA), between 25 °C and 700 °C, with a ramp of 10 °C.min^−1^, in air. The incorporated quantity of polyphenols in HAp NPs was calculated from the weight loss between 250 and 600 °C, assuming that only the release of the polyphenol contributes to the weight loss.

#### 2.4.2. Powder X-ray Diffraction (XRD)

X-ray diffractograms were recorded on a D8 diffractometer (Bruker, Billerica, MA, USA), equipped with a CuKα source (1.54 Å) and a Bragg–Brentano θ–2θ geometry, between 7° and 80° with a variable slit V12, a step of 0.03°, and a scan speed of 1 s/point, except for the diffractogram of HAp obtained in diluted conditions that was recorded with a fast detector LynxEye (Bruker, Billerica, MA, USA) with a variable slit V12 and an exposition time of 1.67/step/canal. Crystallite sizes were calculated using the Scherrer equation. The diffraction peak at 2θ = 26°, corresponding to the plan (002), was used for the calculation due to its adequate resolution and absence of overlap with other peaks.

#### 2.4.3. Transmission Electron Microscopy (TEM)

Nanoparticles were deposited on 300 square mesh carbon-coated copper grids (Ted Pella, Redding, CA, USA) and observed with a Tecnai spirit G2 microscope (Thermofischer Waltham, MA, USA), operating at 120 kV, equipped with a Orius 1000 camera (Gatan AMTEK, Pleasanton, CA, USA).

#### 2.4.4. Nuclear Magnetic Resonance (NMR) Spectroscopy

Solid-state nuclear magnetic resonance (NMR) experiments were performed on a Bruker Avance III spectrometer (Billerica, MA, USA) operating at 300.3, 121.6, and 75.5 MHz for ^1^H, ^31^P, and 13C, respectively. A broadband dual resonance 4 mm probe was used with Magic Angle Spinning (MAS) at νr = 11.962 kHz. 1H experiments were acquired with a 90° pulse, a relaxation delay RD = 2 s, and a number of scans NS = 4. {^1^H}-^31^P spectra were obtained using cross-polarization (CP) with a contact time tcp =1 or 10 ms, RD = 1 s, and NS = 128. For {^1^H}-^13^C experiments, CP was used with tcp =2 ms, RD = 1 s, and NS = 47752. SPINAL-64 decoupling [40] was applied at ν^1^H = 70 kHz. Spectra were referenced to TMS for ^1^H and ^13^C, and to H_3_PO_4_ at 85% for ^31^P.

#### 2.4.5. Textural Properties

N_2_ sorption isotherms were obtained using a Belsorp max instrument (MICROTRAC MRB, Haan, Germany). Specific surface areas were calculated following the Brunauer–Emmett–Teller (BET) method.

### 2.5. Polyphenol Release

A total of 10 mg (except in case of HAp synthesized in the presence of BE where 1 mg of solid was used) of hybrid HAp/polyphenol solid was suspended in 2 mL of PBS buffer (10 mM, pH 7.4) at room temperature. The suspension was stirred by a horizontal axis rotating stirrer. At designed time intervals, the suspension was centrifuged (5 min at 2500 rpm) and 500 µL of the supernatant was taken out for the assay and immediately replaced by 500 µL of fresh PBS buffer to maintain the volume of the supernatant constant. The supernatant was analyzed by HPLC using an Agilent C18 column (Poroshell 120 4.6 × 100 mm, 4µm) maintained at 40 °C. The mobile phase was a equivolume mixture of water and acetonitrile. The amount of released polyphenol was determined by measuring the area of the corresponding peak based on calibration curves. The detection wavelength was 220 and 287 nm for CA and RA, respectively, and 270 nm for both BA and BE. The release experiment of BA was carried out in duplicate and favorable reproducibility was observed.

### 2.6. Antioxidant Activity Assays

The antioxidant property of the polyphenols, in solution or within the hybrid nanoparticles, was evaluated by their radical scavenging activity towards 2,2-diphenyl-1-picrylhydrazyl (DPPH) following a modified Blois method [41,42]. Solutions of polyphenols (final concentration 5 µM) or suspensions of 5.0 ± 0.5 mg of the nanoparticles in a TRIS buffer (pH 7.4) were mixed with an equal volume of DPPH solution in ethanol (100 μM). Samples were left to react for 20 min in the dark, centrifuged when required, and absorbance (A) was measured at 517 nm using a Uvikon XS spectrophotometer (Secomam, Ales, France). Absorbance (A_ref_) of a 1:1 mixture of TRIS and DPPH at the same wavelength was used as the reference. Bleaching rate was calculated as:

Bleaching rate (%) = 100 × [1 − (A/A_ref_)] (1)

All experiments were performed in duplicate.

## 3. Results

### 3.1. Hybrid Nanoparticles via Adsorption

**Figure 2:**
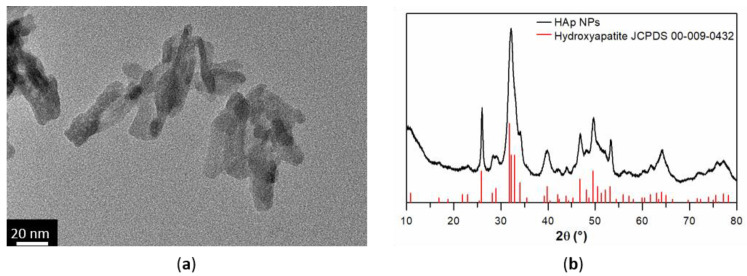
Synthesized HAp NP characterization. (**a**) TEM image; (**b**) X-ray diffractogram.

Hydroxyapatite nanoparticles (HAp NPs) were synthesized by co-precipitation of calcium hydroxide and ammonium dihydrogenophosphate [34]. The reaction yield was 90% based on weight measurements. TEM observation indicated that particles were platelet-like-shaped, 31 ± 6 nm in length, and 9 ± 3 nm in width (Figure 2a). Specific surface area calculated from N_2_ sorption was 160 m² g^−1^ (Figure S1 in Supplementary Materials). The X-ray diffractogram confirmed the formation of hydroxyapatite (Figure 2b).

These HAp nanoparticles were suspended in polyphenol solutions (0.5 mM) for 24 h. The number of adsorbed polyphenols was measured by TGA (Table 1 and Figure S2). BE was the most efficiently adsorbed polyphenol, with 63 mg per g of hybrid particles, loadings in RA and CA were about two times smaller and BA exhibited an intermediate value (37 mg g^−1^). When calculated as the molecular surface density, polyphenol loading was three times higher for BE than for the three other molecules.

**Figure 3:**
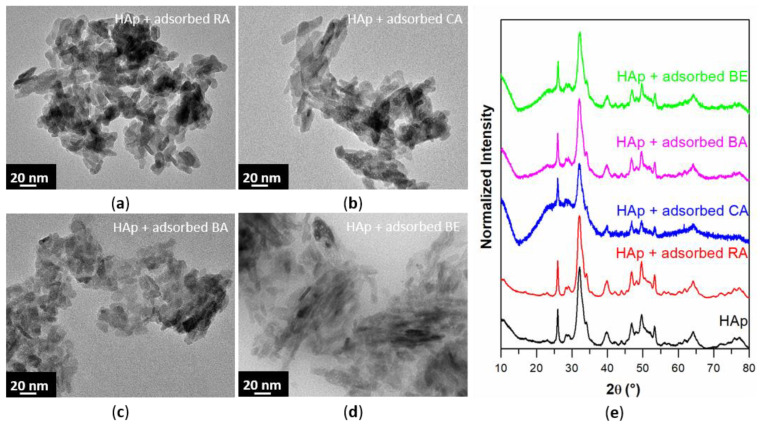
Hydroxyapatite particles after adsorption of polyphenol. TEM images of HAp NPs after adsorption of (**a**) rosmarinic acid; (**b**) chlorogenic acid; (**c**) baicalin; (**d**) baicalein. (**e**) Superimposition of X-ray diffractogram of HAp NPs before and after adsorption of polyphenols. The observed decrease in signal-to-noise ratio in presence of BA, BE, and Ca can be attributed to the low quantity of powder used for the XRD measurement.

TEM did not evidence modification of particle dimension after adsorption (Figure 3a–d and Table 1). X-ray diffractograms did not indicate the formation of additional mineral phases (Figure 3e), and crystallite sizes, as estimated by the Scherrer formula, were similar to the one before adsorption (Table 1).

**Figure 4:**
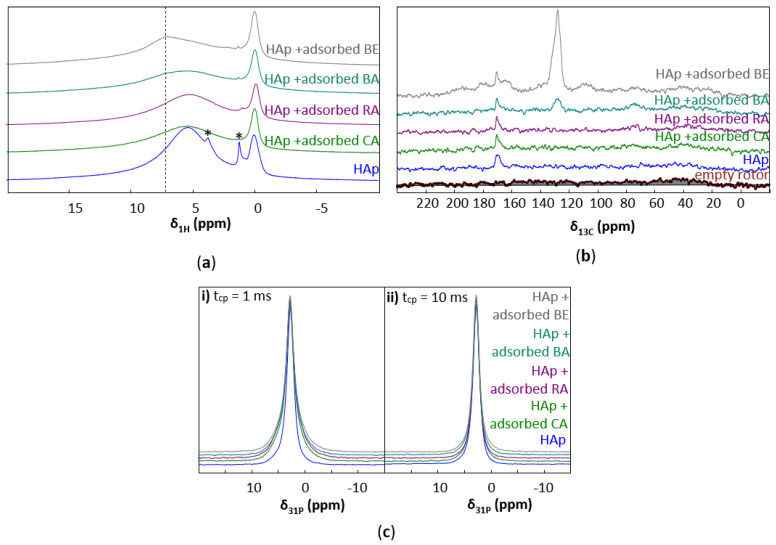
Solid-state NMR experiments performed on pure HAp NPs, HAp-RA, HAp-CA, HAp-BA, and HAp-BE (initial concentration for adsorption = 0.05 mM) (* indicate residual peaks of acetone and ethanol). (**a**) ^1^H; (**b**) {^1^H}-^13^C CP; (**c**) {^1^H}-^31^P CP spectra at tcp = (i) 1 and (ii) 10 ms.

For further characterization, ^1^H, {^1^H}-^13^C, and {^1^H}-^31^P solid-state MAS NMR experiments were performed before and after adsorption. Superimposition of ^1^H spectra is presented in Figure 4a. On the spectrum of pure HAp, the signal at 0 ppm, characteristic of protons in the hydroxyapatite crystal lattice, could be noticed, together with a broader peak at 5.5 ppm, which can be ascribed to physisorbed water [43]. Similar spectra were obtained for HAp-RA, HAp-CA, and HAp-BA, except that the peak at 5.5 pm was broader. For HAp-BE, this peak was not clearly identified. Instead, a broad band was observed at 7.2 ppm, which can be attributed the aromatic ring of this molecule.

Spectra obtained by {^1^H}-^13^C CP experiments are presented in Figure 4b. A residual rotor background signal was present, as shown in gray on the bottom spectrum. All spectra were characterized by a broad peak of weak intensity at 170 ppm ascribed to carbonate species (Figure 4b). This band is dissymmetrical, which suggests a type B substitution, i.e., a substitution of phosphate groups by carbonate groups in the crystal lattice of hydroxyapatite [44]. In addition, an intense peak was visible at approximatively 128 ppm on the HAp-BE spectrum, and, to lesser extent, on the one of HAp-BA. This band corresponds to aromatic carbons of baicalein and baicalin.

{^1^H}-^31^P CP spectra at tcp = 1 and 10 ms are presented on Figure 4c (i) and (ii), respectively. At tcp = 1 ms, both crystalline (narrow) and amorphous (broad) components were visible at 2.8 ppm. For all samples, adsorption leads to a broadening of this peak, highlighting an increase in the relative amount of the amorphous component. At a longer contact time (tcp = 10 ms), all spectra were identical, showing no significant modification of the structure of the core of HAp particles.

### 3.2. Hybrid Nanoparticles via In Situ Incorporation

**Figure 5:**
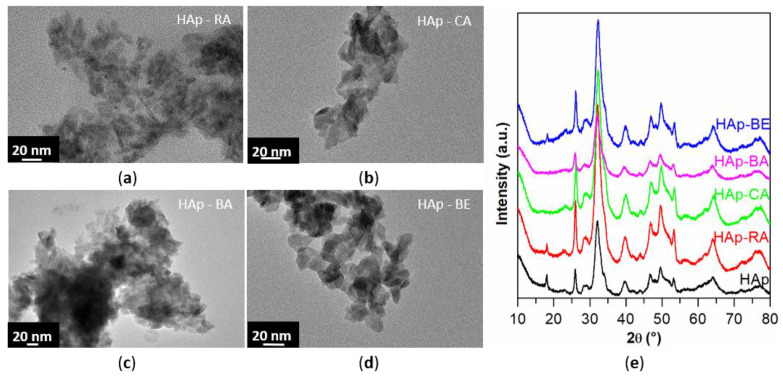
Hydroxyapatite particles synthesized in presence of 0.05 mM polyphenols. (**a**) Superimposition of X-ray diffractogram of HAp NPs, HAp-RA, HAp-CA, HAp-BA, and HAp-BE; TEM images of (**b**) HAp-RA; (**c**) HAp-CA; (**d**) HAp-BA; (**e**) HAp-BE.

In a first step, it was first necessary to check whether the use of lower calcium hydroxide and ammonium dihydrogenophosphate concentrations impacted HAp particle structure. XRD showed that although hydroxyapatite was the major recovered phase, an additional peak at ca. 2θ = 18° was present (Figure 5a). This diffraction peak could be assigned to the (001) diffraction peak of Ca(OH)_2_, although this assumption could not be fully confirmed due to the overlap between Ca(OH)_2_ and HAp patterns for all other calcium hydroxide peaks.

In the presence of polyphenols at 0.05 mM, formation of hydroxyapatite was assessed by XRD (Figure 5a). Only the peaks assigned to HAp were observed in the presence of BA and CA while the additional peak at ca. 2θ = 18° was visible with RA and BE. The calculated crystallite size was similar for all samples. TEM images showed that HAp-CA and HAp-BA consisted of platelet-shaped particles, similar to pure HAp, whereas HAp-RA and HAp-BE particles had a less defined shape (Figure 5b–e). The length of the particles was reduced from 30 ± 7 nm for pure HAp to 14 ± 2 nm for HAp-RA, 19 ± 2 nm for HAp-CA, 13 ± 2 nm for HAp-BA, and 17 ± 3 nm for HAp-BE (Table 2). In parallel, particle width remained unchanged compared to pure HAp NPs (8 ± 4 nm) in the presence of CA and BE but significantly decreased with RA and BA (4.2 ± 0.5 nm) (Table 2).

The presence of polyphenols also influenced the reaction yield, as calculated from the mass of dry solid, from ca. 65% for HAp NPs alone to 45–55% in the presence of the four molecules (Table 2). TGA measurements indicated that polyphenol loading was 52 mg·g^−1^ for RA and BE, 42 mg·g^−1^ for CA, and 54 mg·g^−1^ for BA (Table 2 and Figure S3). The final polyphenol-to-calcium molar ratio in the hybrid particles ranged between 0.01 for HAp-BA and 0.02 for HAp-BE, thus being higher than in the initial solution (0.0075) (Table S1).

**Figure 6:**
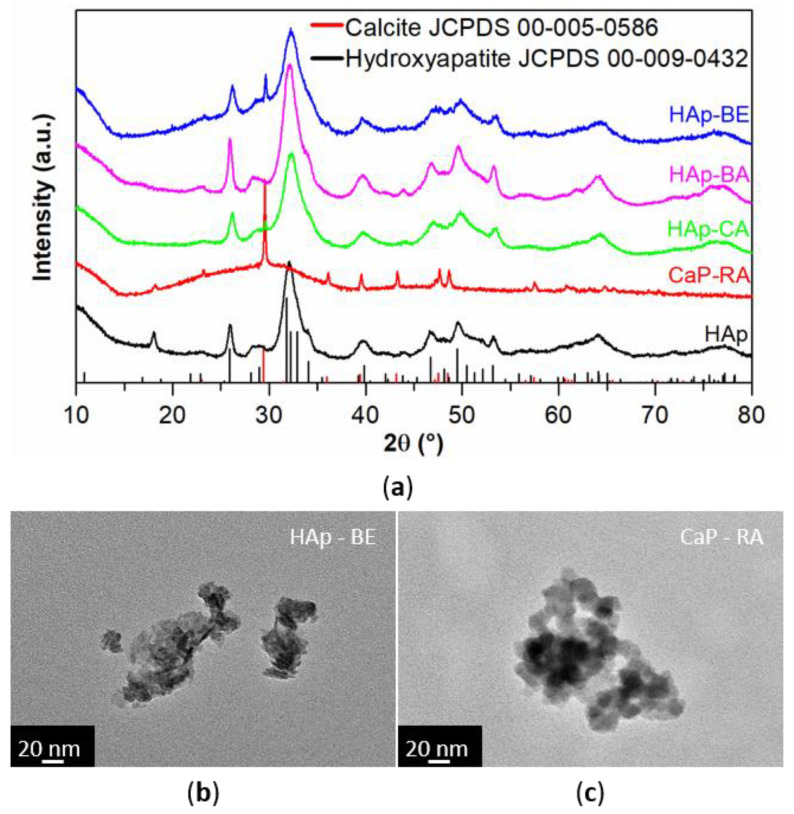
Hydroxyapatite particles synthesized in presence of 0.5 mM polyphenols. (**a**) Superimposition of X-ray diffractogram of HAp NPs, CaP-RA, HAp-CA, HAp-BA, and HAp-BE compared to patterns of hydroxyapatite (JCPDS 09-0432) and calcite (JCPDS 0.5-0586); TEM images of (**b**) HAp-BE; (**c**) CaP-RA.

A second set of experiments was performed at a 0.5 mM polyphenol concentration in an attempt to further increase their content in the particles. Such an increase in the initial concentration of BA and BE was possible because of their enhanced solubility at pH 12 (compared to the adsorption conditions performed in deionized water). XRD patterns presented in Figure 6a show that hydroxyapatite was the main precipitated mineral phase in the presence of CA, BE, and BA. Calculated crystallite sizes were similar to the ones obtained in the presence of 0.05 mM polyphenols, ca. 15 nm (Table 2).

In contrast, synthesis of HAp NPs in the presence of 0.5 mM RA was unsuccessful. The particle washing step in distilled water led to the dissolution of the solid phase obtained by co-precipitation so that almost no solid could be harvested after this step. In order to recover a few mg of material to perform XRD analysis, the washing step was skipped. The X-ray diffractogram of this raw sample did not show peaks corresponding to the hydroxyapatite structure but comparison of experimental data with the database showed that the pattern could be assigned to the calcium carbonate (CaCO_3_) calcite phase. HAp formation was thus totally inhibited by the presence of rosmarinic acid. Therefore, this compound was referred as CaP (for calcium phase)-RA. The identification of CaP-RA as calcite is consistent with its solubilization during washing, as calcite is known to be much more soluble in water near neutral pH than in alkaline conditions [45]. The morphology of CaP-RA was found to be very different from other HAp NPs since particles were larger and rectangular-shaped whereas HAp-BE particles were platelet-like-shaped (Figure 6b,c, respectively). It is worth noting that the main diffraction peak of calcite (2θ = 29°) is also visible on the diffractogram of HAp-BE and, to a lesser extent, that of HAp-CA but not HAp-BA.

For HAp-CA and HAp-BE NPs, the apparent reaction yield in the presence of a 0.5 mM polyphenol was lower than with 0.05 mM, while it was similar for HAp-BA at both concentrations (Table 2). The number of incorporated polyphenols was 73 mg·g^−1^ for CA, 135 mg·g^−1^ for BE, and 111 mg·g^−1^ for BA, based on TGA analysis. An increase in the initial concentration thus allowed for the incorporation of more CA, BA, and BE in the material, BE being the most efficiently incorporated molecule. However, whereas the initial polyphenol solutions were 10 times more concentrated, the final incorporated quantity was not 10 times higher; loadings at 0.5 mM were only twice those obtained at 0.05 mM. Moreover, no enrichment of the solid compared to the initial solution was observed as the polyphenol-to-calcium ratio in the solid was smaller than the initial solution, i.e., 0.075 (Table S1).

### 3.3. Polyphenol Release

**Figure 7:**
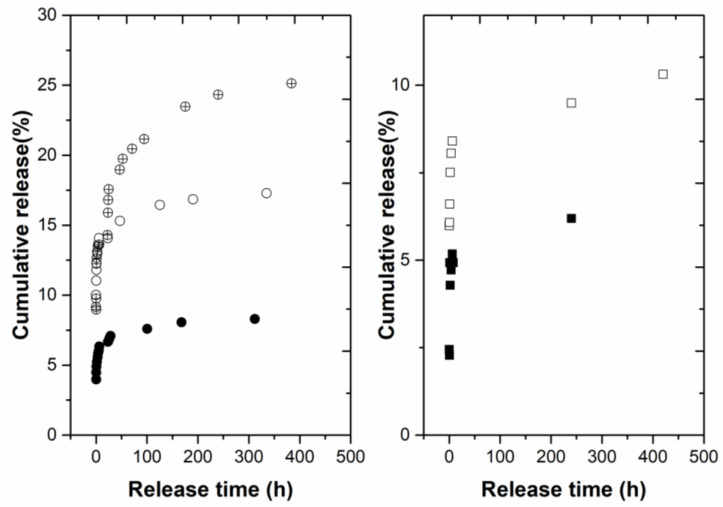
Cumulative release of baicalin (left Figure) and baicalein (right Figure). Empty symbols for adsorbed flavonoids and filled symbols for in situ incorporated ones (at 0.05 mM polyphenol initial concentration). Crossed open circles correspond to baicalin release after replacing full medium with fresh PBS after 22 h.

In order to evaluate the potential drug delivery ability of hybrid HAp particles, the release of immobilized polyphenols was studied. Figure 7, (left) represents the cumulative release profile of BA from HAp NPs in PBS (pH 7.4). From observing the plot, it is obvious that BA release has a burst effect during the first six hours, amounting for 14% of adsorbed BA and 7% of in situ incorporated BA. For longer release times, the rate of BA release became slower and the cumulative release after ca. 2 weeks was 17% and 8% for adsorbed and in situ incorporated BA, respectively. It is worth noting that, when the totality of the supernatant was replaced by fresh PBS after 22 h of release, an additional fast release step was observed and a cumulative release of 25% could be reached for BA. A very similar behavior to BA was observed for BE (Figure 7, right), with a very fast initial release rate during the first 6 h and a significantly slower rate over longer durations. However, overall, BE was less released since no more than a 10% and 6% cumulative release was reached after 419 and 319 h for adsorbed and in situ incorporated BA, respectively. For both BA and BE, it can be noticed that the cumulative release was higher when the flavonoid was adsorbed on HAp compared to in situ incorporated.

**Figure 8:**
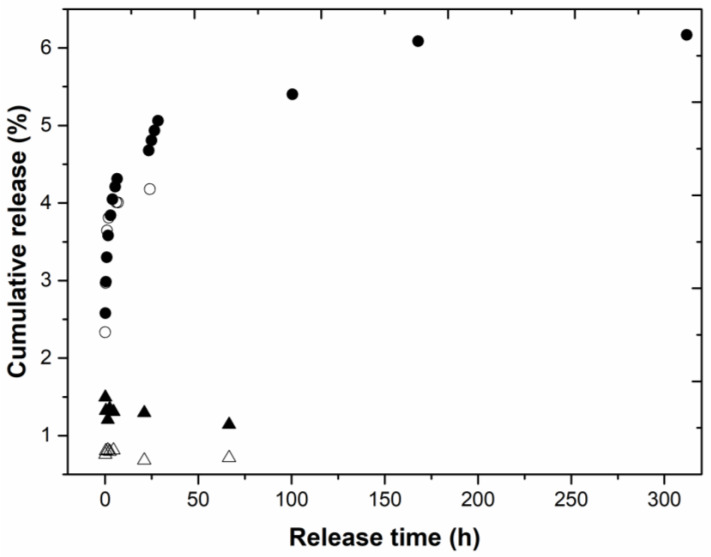
Cumulative release of rosmarinic acid (open circles, adsorbed on HAp; solid circles, incorporated in situ) and chlorogenic acid (open triangles, adsorbed on HAp; solid triangles, incorporated in situ at 0.05 mM polyphenol initial concentration).

Rosmarinic acid showed a similar release profile during the first 6 h of release, regardless of its immobilization method, reaching a cumulative release of 4% (Figure 8). In an attempt to further increase the adsorbed RA release, the totality of the supernatant was replaced by fresh PBS solution 7 h after the beginning of the release process. However, after 24 h, the cumulative release only reached 4.2%. When RA was in situ incorporated in HAp, the release rate remained sustained until 28 h and then decreased to reach a 6% cumulative release after 312 h. For the CA results, it is worth noting that its cumulative release remained very low, below 1.5%, regardless of the immobilization method.

### 3.4. Anti-Radical Properties

**Figure 9:**
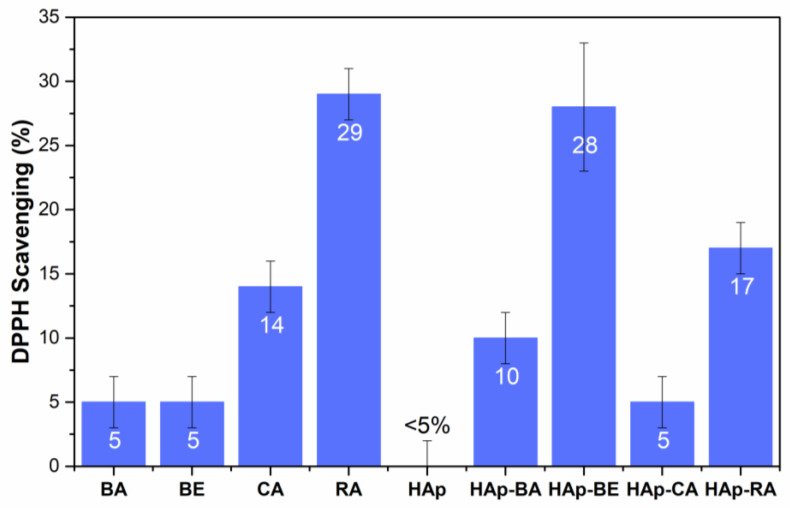
Antioxidant activity. DPPH scavenging ability percentage of polyphenols in solution and after adsorption on hydroxyapatite (HAp) particles, after 20 min of reaction.

The antioxidant properties of the molecules were evaluated in solution, after adsorption on HAp NPs and after in situ incorporation (at 0.05 mM), by measuring their anti-radical abilities. This was performed using the Blois method based on the spectrophotometric monitoring of the bleaching of a DPPH solution after 20 min of reaction [41,42]. As shown in Figure 9, the measured capacity to scavenge DPPH radicals in these conditions ranged from ca. 30% to ca. 5% in the following decreasing order: RA > CA > BA = BE. After adsorption, the DPPH bleaching range was similar while HAp NPs alone produced a negligible bleaching of DPPH solution. However, the order was modified to BE > RA > BA > CA. Finally, hybrid particles formed by in situ incorporation all only have a negligible ability to scavenge DPPH radicals.

## 4. Discussion

We have assessed two methods to prepare hybrid NPs, combining four natural polyphenols, rosmarinic acid, chlorogenic acid, baicalin, and baicalein with hydroxyapatite, either by adsorption or in situ incorporation. The adsorption process occurred without significant modification of the HAp nanoparticles’ shape or structure, regardless of the polyphenol tested. The second method allowed us to obtain hybrid nanoparticles for all polyphenols, except RA when used at 0.5 mM. However, the reaction yield was decreased and calcite formation was evidenced, except in the presence of BA.

Adsorption of biologically active molecules on HAp NPs have often been reported in the literature. For instance, antibiotics, such as gentamicin or tobramycin but also polyphenols, as extracts or single molecules, have been adsorbed on hydroxyapatite nanoparticles or coatings [36,46,47,48,49]. The adsorption of bisphosphonates on hydroxyapatite nanoparticles have also been described, conferring osteogenic properties to the materials [23,24].

Three main kinds of interactions can be expected between molecules and HAp surfaces: (1) electrostatic, (2) hydrogen bonding, and (3) π-cation interaction between aromatic ring of polyphenols and Ca^2+^ in HAp [50]. The isoelectric point (IEP) of hydroxyapatite can range between 7 and 8 [51,52], so that the HAp surface could be weakly positively charged in the sorption conditions of this work. pKa of the carboxylic acid of RA, CA, and BA is 2.78, 3.90, and 2.8, respectively [13]. RA, CA, and BA are thus negatively charged in the adsorption experimental conditions (pH < 7), so that attractive electrostatic interactions could drive their adsorption on the HAp surface. However, although BE is not fully deprotonated at pH 5 (first pKa for the phenol group is 5.5 ± 0.1 [53,54]), it is the most adsorbed polyphenol: on a molecular basis, BE was three times more adsorbed than BA, RA, and CA on HAp. This is consistent with a study by Chirdon et al. [32] that showed that catechol and pyrogallol derivatives adsorbed more readily and strongly on HAp surfaces than phenol and carboxylic acids. The proposed explanation is based on cooperative hydrogen-bond interactions via the catechol or pyrogallol groups of the adsorbed molecule and HAp surface. To gain further insight into the nature of adsorption of catechol on HAp, the authors calculated that the apparent occupied area for each catechol molecule was 3.8 nm^2^. It is worth mentioning that this value is very close to the one calculated here for RA, CA, and BA from experimental data, i.e., 3.3 nm^2^. However, Chirdon et al. pointed out that their area is far greater than the theoretical area per molecule, which is approximately 0.067 nm^2^, thus suggesting a cooperative binding of catechol through divalent hydrogen bonds on the HAp crystal edges.

In the present work, the fact that BA is less efficiently adsorbed than BE shows that the loss of one available OH group on the pyrogallol ring is not compensated by the presence of an additional carboxylate group on the sugar moiety. Accordingly, the catechol ring present on CA and RA is less efficient than the pyrogallol ring of BE to interact with the HAp surface. This is also supported by the difference in acidity between the pyrogallol ring of BE, whose pKa is around 5.5, and the catechol rings of BA, RA, and CA whose pKa are above 8 [55,56]. In the sorption conditions, only the pyrogallol ring of BE is partially deprotonated, explaining its stronger affinity for the HAp surface compared to the other studied polyphenols. Such a complex pH-dependent process with multiple adsorption mechanisms was also emphasized with tannic acid adsorption on HAp since tannic acid contains both pyrogallol and carboxylic groups [57].

Coming to the in situ approach used in the present work, the synthesis of hydroxyapatite in the presence of polyphenols was successfully performed for an initial polyphenol-to-calcium molar ratio of 0.0075. It led to hybrid nanoparticles of a smaller size than in the absence of polyphenols while their crystallite sizes remained unchanged, showing no inhibitory effect of the polyphenols on crystal growth. The relative number of polyphenols in the solid, as determined by TGA, increased by around 60% for a given molecule compared to the adsorption method, except for BE. In that case, the in situ incorporation method led to a decrease in the immobilized BE by 17%. However, ca. 5.2 wt% of BE was found in the solid. It is worth noting that a similar relative amount (3.1 wt%) was found in HAp nanocrystals prepared in the presence of the flavonoid quercetin, according to a precipitation method at a basic pH [21]. Vasquez et al. also succeeded in the tannic acid-assisted synthesis of HAp nanoparticles. However, in that case, the amount of incorporated tannic acid was not determined [58].

Electrostatic interactions leading to the formation of complexes between polyphenol and Ca^2+^ could be expected when using the in situ method since it involves a preliminary step where Ca(OH)_2_ is dissolved in the presence of the polyphenols, which are negatively charged at pH 12. Except for BE, electrostatic interactions are likely to take place between calcium ions and polyphenols through their carboxylic group. Additionally, phenolic groups are also deprotonated at pH 12 and can also interact with calcium ions. However, it has been fully established that catechol groups are not stable in alkaline conditions and undergo a redox transition to quinones upon an increase in pH value [59,60]. Here, this oxidation process was confirmed by UV-Vis spectroscopy (Figure S4) showing a clear decrease in the absorbance intensity of bands representative of aromatic hydroxyl groups. However, as the kinetics of the catechol oxidation process is rather low (after one hour, the characteristic band of the aromatic ring of BE around 370 nm is still visible (Figure S4)), it can be assumed that BE can interact with Ca^2+^ and influence the HAp precipitation mechanism before its complete oxidation. When increasing the initial polyphenol-to-Ca molar ratio by a factor of 10, it was possible to incorporate more polyphenols in the HAp for BA, BE, and CA with a small decrease in reaction yield but without impacting crystallite size. However, only twice the amount of BA, BE, and RA was measured by thermogravimetry, suggesting the existence of a maximum loading.

However, in the presence of rosmarinic acid with a polyphenol-to-calcium molar ratio of 0.075, the formation of hydroxyapatite was fully inhibited. Such an inhibition was previously reported for other natural polyphenols, such as gallic acid and tannic acid, but the underlying mechanisms were not studied in detail [37,38]. Besides interactions with calcium ions in solution, RA may influence other steps of the HAp formation process. To start with, they may interfere with calcium speciation. To check this hypothesis, the time evolution of a Ca(OH)_2_ suspension in the absence or presence of RA was followed by XRD measurements (Figure S5). In the absence of RA, after 15 min, a very low amount of solid could be recovered, suggesting fast dissolution of calcium hydroxide. Such a result is consistent with the solubility value of calcium hydroxide in water, 1.48 g·L^−1^ at 25°C [59], whereas the calcium hydroxide concentration after its complete dissolution was expected to be 0.74 g·L^−1^ under the chosen experimental conditions. After 30 min, the solid content recovered from the solution increased and XRD indicated that it consists of pure CaCO_3_. Formation of calcite in these conditions is indeed thermodynamically favored [61,62]. A similar dissolution process of Ca(OH)_2_ was observed in the presence of 0.05 mM and 0.5 mM of RA, except that in the latter case, traces of solid Ca(OH)_2_ were detected in addition to calcite. These would indicate that RA has only a minor influence on calcium speciation in the early stages of HAp formation.

**Scheme 1:**
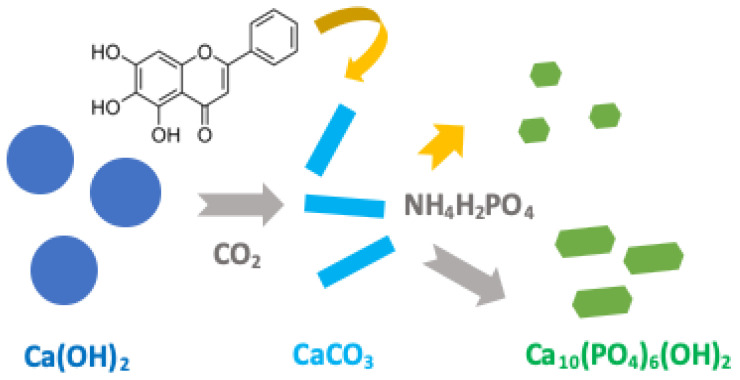
Formation of hydroxyapatite nanoparticles. The formation of hydroxyapatite nanoparticles occurs by conversion of calcium hydroxide into calcite by ambient CO_2_ followed by reaction with ammonium dihydrogenophosphate yielding to hydroxyapatite. When polyphenols are initially present, they may adsorb on the calcite intermediate particles, partially or fully inhibiting hydroxyapatite formation.

In the absence of RA or in the presence of 0.05 mM RA, HAp was formed, while in the presence of RA at 0.5 mM, CaCO_3_ was the only solid identified by XRD. Synthesis with BE, BA, and CA led to intermediate situations with both CaCO_3_ and HAp being present. RA at 0.5 mM may therefore inhibit CaCO_3_ dissolution and, consequently, HAp formation. In fact, TEM images show that all polyphenols induce a decrease in HAp particle size, which could be a consequence of a growth inhibition of HAp. Such a growth limitation can result either from adsorption of the polyphenols on the surface of growing particles of HAp or a depletion in available Ca^2+^. The first possibility is very unlikely because the most efficiently adsorbed polyphenol on pre-formed HAp NPs is BE while this molecule did not show a particular inhibiting effect on HAp crystal growth in this study. Accordingly, RA did not exhibit any specifically high affinity for the HAp surface but it has the highest inhibiting action on precipitation. Altogether, this would favor the hypothesis that the underlying mechanism is mainly related to polyphenol interactions with CaCO_3_ that would limit its dissolution (Scheme 1). The latter hypothesis is in line with previous reports showing that some carboxylic derivatives at low concentrations (less than 0.01 mol·L^−1^) can inhibit the dissolution of CaCO_3_ in alkaline pH [63,64]. Inhibition was attributed to the blocking of dissolution sites by adsorbed carboxylates. Among the tested polyphenols, RA, with one carboxylate and two catechol functions, may thus be the most efficiently adsorbed on CaCO_3_, preventing its dissolution. Noticeably, Forte et al. reported that quercetin partially inhibited HAp formation in basic conditions, as indicated by a reduction in crystallite size [21]. In acidic conditions, quercetin allowed for the formation of monetite (CaHPO_4_) but inhibited the monetite-to-hydroxyapatite transformation in basic conditions at a high concentration. This confirms that HAp formation inhibition can result from interaction of molecules with intermediate phases rather than with precursor salts.

Finally, release experiments showed that adsorbed baicalin was the most released polyphenol with up to 17% of cumulative release after 2 weeks. It is worth noting that the release conditions are close to the ones of the polyphenol adsorption experiments at equilibrium and that the polyphenol concentration in the supernatant during the initial phase of the release kinetics are very close to the ones of the adsorption step at equilibrium. Moreover, it was possible to enhance the baicalin release rate after 22 h by removing the totality of the supernatant. Altogether, these results show that the adsorption process of baicalin is at least partly reversible and could lead to the release of around 40 µM of baicalin under the chosen experimental conditions. For both baicalin and baicalein, the release was slower when the polyphenol was in situ incorporated than when adsorbed on NPs, which is consistent with the molecular diffusion limitation process in the latter case.

However, such an argument is not relevant in case of rosmarinic acid and chlorogenic acid since the release kinetics was identical for both adsorbed and in situ incorporated polyphenols during the first 6 h. Noticeably, for RA, cumulative release for in-situ-loaded particles was comparable to BA and BE (ca. 6%), while that for adsorbed NPs was smaller (ca. 4%) and could not be enhanced by total replacement of the supernatant after 6 h. In the case of CA, the cumulative release was less than 1%, with a significant experimental error because of the very low signal-to-noise ratio observed for the corresponding chromatograms. This would suggest that, in contrast to BA and BE, RA and CA adsorption on the HAp surface has a significant irreversible character. This could result from the presence of a C=C bridge in their molecular structure, conferring them conformational flexibility and favoring their reorganization to maximize their interaction with the HAp surface [65].

As a first evaluation of the antioxidant efficiency of the hybrid material, their anti-radical properties were studied using Blois method based on the bleaching of a DPPH radical solution. The time interval between mixing the samples, DPPH, and reading the absorbance is critical for interpreting the results of this assay [66]. In this work, absorbance was read after 20 min of reaction. Considering that the kinetics of the reaction for polyphenols in a polar solvent is fast, due to the participation of fast electron transfer in the mechanism of anti-radical activity [53,66], we can assume that the reaction reached its equilibrium for the four tested molecules in these conditions and we can link the anti-radical activity of the hybrid particles to their antioxidant properties. Furthermore, the concentration of polyphenols is very important as the mechanism is concentration-dependent [53]. Here, the anti-radical ability of hybrid particles was compared so that the concentration of particles in suspension was fixed. Gaining further insight into the activity of the immobilized polyphenols would require additional studies with a fixed molecular content. Our measurements showed that the in situ approach led to inactive nanomaterials. This agrees with the above-suggested autoxidation of polyphenols to quinones in the alkaline pH conditions of the in situ synthesis. However, an alternative and possibly additional explanation is that polyphenols are incorporated inside the nanoparticles and are therefore not accessible to DPPH. In contrast, all hybrid particles obtained by adsorption showed some antioxidant activities. It is worth mentioning that, due to experimental constraints, the concentration of polyphenols in particle suspension is significantly larger than in the polyphenol solutions, so that bleaching of DPPH cannot be compared between these two sets of experiments. The most DPPH bleaching was obtained either for high loadings of BE with a low intrinsic activity or low loadings of RA with a high intrinsic activity. This strongly suggests that further enhancing the number of adsorbed polyphenols, for example, using mesoporous nanoparticles exhibiting high specific surface area, would allow us to obtain nanomaterials with strong antioxidant properties.

Finally, our results emphasize the valorization of natural bioactive molecules in nanomedicine, particularly through their release from hydroxyapatite nanoparticles. As previously reported in the literature [67], HAp NPs tend to aggregate in aqueous media, such as PBS, and in biological media. Nevertheless, this phenomenon would not interfere with their internalization by cells that is commonly described as occurring via endocytosis [67,68]. Since HAp is highly insoluble in physiological conditions but has a significant solubility in acidic conditions [69], it is expected that most polyphenols will be released in the acidic intracellular compartments.

## 5. Conclusions

The feasibility of associating natural polyphenols with hydroxyapatite nanoparticles synthesized by precipitation in aqueous medium was established. The in situ incorporation approach was successful in most tested conditions, but the presence of organics lowered the reaction yield or even totally inhibited hydroxyapatite formation. This was attributed to the ability of polyphenols to inhibit the dissolution of CaCO_3_, an intermediary phase in the precipitation reaction. Moreover, resulting particles showed no antioxidant activity, probably due to polyphenol degradation under selected synthetic conditions. On the contrary, post-synthesis adsorption did not modify the size of hydroxyapatite particles, leading to hybrid nanoparticles with antioxidant properties. However, polyphenol loading was limited by their poor solubility in aqueous solutions. Considering the wide variety of plant-extracted polyphenols exhibiting therapeutic properties, these results provide useful guidelines for the improved integration of natural bioactive molecules within nano-biomaterials.

## Figures and Tables

**Table 1:** Adsorption of polyphenols on HAp NPs. Quantity of adsorbed polyphenol on HAp measured by TGA, particle dimensions as obtained from TEM, and crystallite sizes from XRD.

Sample	Incorporated Amount (mg·g^−1^)	Incorporated Amount (Molecules· nm^−2^)	Particle Length/Width (nm)	Crystallite Size (nm)
HAp-RA	31	0.3	31 ± 10/8 ± 2	21 ± 5
HAp-CA	27	0.3	43 ± 11/12 ± 1	23 ± 5
HAp-BA	37	0.3	27 ± 4/8 ± 3	24 ± 5
HAp-BE	63	0.9	33 ± 10/15 ± 3	24 ± 5
HAp	-	-	31 ± 6/9 ± 2	21 ± 2

**Table 2:** Synthesis of HAp NPs in presence of polyphenols at 0.05 mM and 0.5 mM. Yield, particle dimensions as measured from TEM, crystallite sizes calculated from XRD data, incorporated quantity measured from TGA.

Sample	Initial Concentration (mM)	Yield (%)	Particle Length/Width (nm)	Crystallite Size (nm)	Incorporated Quantity (mg· g^−1^)
HAp-RA	0.05	46	14 ± 2/4.2 ± 0.5	16 ± 1	52
	0.5	-	-	-	-
HAp-CA	0.05	56	19 ± 2/6 ± 2	15 ± 1	42
	0.5	43	-	14 ± 2	73
HAp-BA	0.05	46	13 ± 2/4.2 ± 0.5	23 ± 2	54
	0.5	50	-	18 ± 2	111
HAp-BE	0.05	53	17 ± 3/7 ± 1	17 ± 1	52
	0.5	42	-	14 ± 3	135
HAp	-	66	30 ± 7/8 ± 4	15 ± 3	-

## Data Availability

Data are available from the authors upon reasonable request.

## Supplementary Materials

The following supporting information can be downloaded at: https://www.mdpi.com/article/10.3390/nano12203588/s1, Figure S1: N_2_ sorption isotherm of hydroxyapatite nanoparticle powder; Figure S2: TGA analyses of hydroxyapatite nanoparticles before and after adsorption of polyphenols; Figure S3: TGA analyses of hydroxyapatite nanoparticles synthesized in presence of polyphenols; Figure S4: Stability of (a) RA, (b) CA, (c) BE, and (d) BA at pH 12; Figure S5: XRD of powder recovered after 30 min in solution of Ca(OH)_2_ and Ca(OH)_2_ + RA, and comparison with XRD of calcite (PDF card JCPDS 05-0586); Table S1: Synthesis of HAp NPs in presence of polyphenols at 0.05 mM and 0.5 mM; Yield, particle dimensions as measured from TEM, crystallite sizes calculated from XRD data, incorporated quantity measured from TGA and estimated final polyphenol-to-Ca molar ratio.
