# Supplementary material for: Synthesis of Hybrid Polyphenol/Hydroxyapatite Nanomaterials with Anti-Radical Properties
Source: Nanomaterials (Basel). 2022 Oct 13;12(20):3588. doi: 10.3390/nano12203588 (PMC9612319; doi:10.3390/nano12203588)
Supplement: Supplementary file 1 [file nanomaterials-12-03588-s001.zip › nanomaterials-1938402-supplementary.pdf]

Supporting Information

Synthesis of Hybrid Polyphenol/Hydroxyapatite Nanomaterials with Anti-Radical Properties

Estelle Palierse [1,2], Sylvie Masse [1], Guillaume Laurent [1], Patrick Le Griel [1], Gervaise Mosser [1], Thibaud Coradin [1,*] and Claude Jolivalt [2,*]

[1] Sorbonne Université, CNRS, Laboratoire de Chimie de la Matière Condensée de Paris, 75005 Paris, France
[2] Sorbonne Université, CNRS, Laboratoire de Réactivité de Surface, 75005 Paris, France
* Correspondence: thibaud.coradin@sorbonne-universite.fr (T.C.); claude.jolivalt@sorbonne-universite.fr (C.J.)

Figure S1. N_2 sorption isotherm of hydroxyapatite nanoparticle powder.

Figure S2. TGA analyses of hydroxyapatite nanoparticles before and after adsorption of polyphenols.

Figure S3. TGA analyses of hydroxyapatite nanoparticles synthesized in presence of polyphenols.

Figure S4. Stability of (a) RA, (b) CA, (c) BE and (d) BA at pH 12.

Figure S5. XRD of powder recovered after 30 min in solution of Ca(OH)2 and Ca(OH)2 + RA, and comparison with XRD of calcite (PDF card 05-0586).

Table S1. Synthesis of HAp NPs in presence of polyphenols at 0.05 mM and 0.5 mM: yield, particles dimensions as measured from TEM, crystallite size calculated from XRD data, incorporated quantity measured from TGA and estimated final polyphenol: Ca molar ratio.

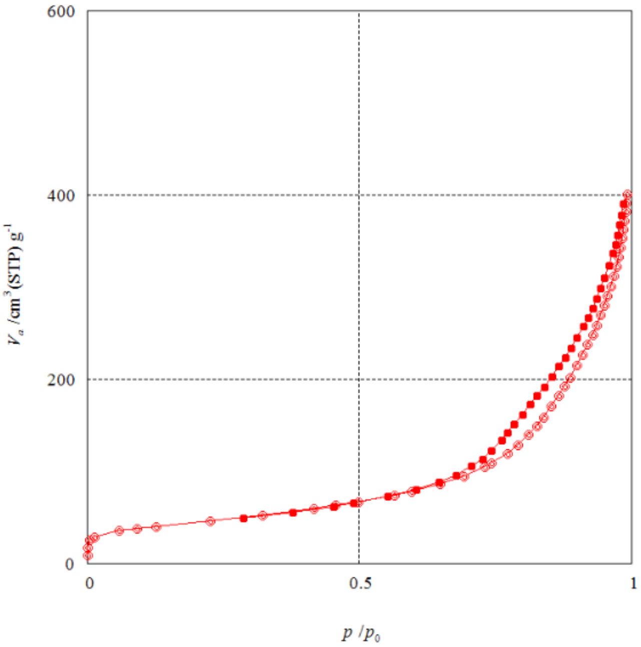

Figure S1. N_2 sorption isotherm of hydroxyapatite nanoparticle powder.

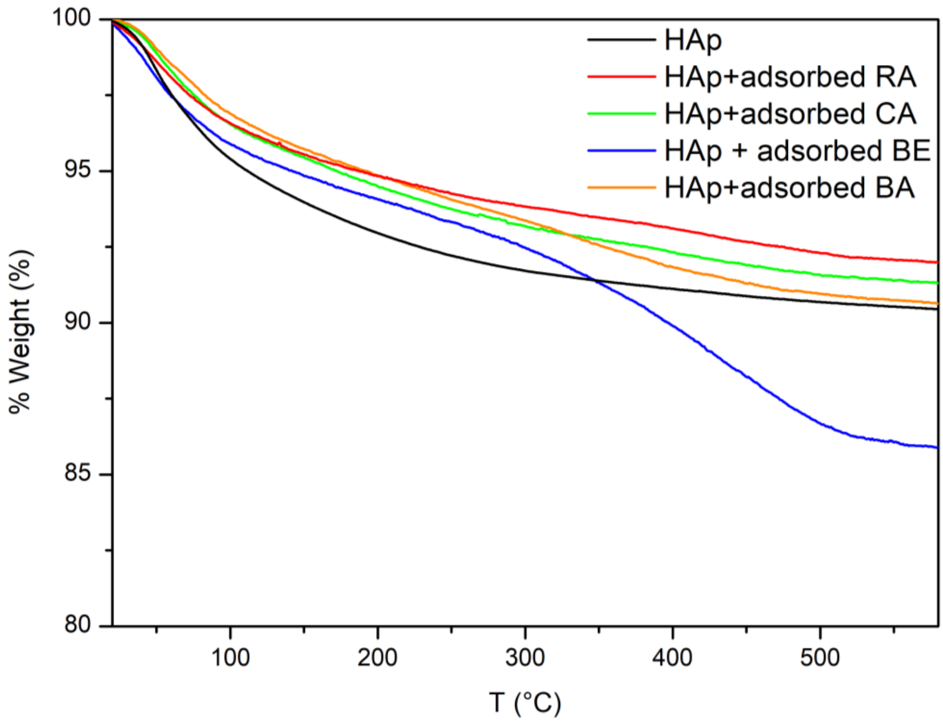

Figure S2. TGA analyses of hydroxyapatite nanoparticles before and after incorporation of polyphenols.

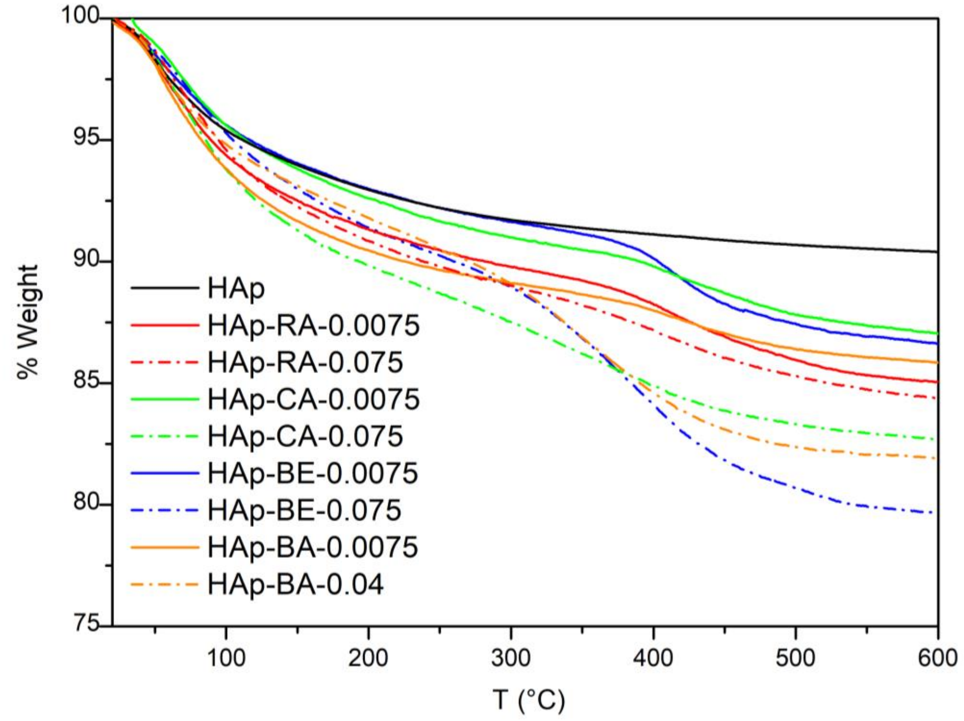

Figure S3. TGA analyses of hydroxyapatite nanoparticles when polyphenols are incorporated during synthesis.

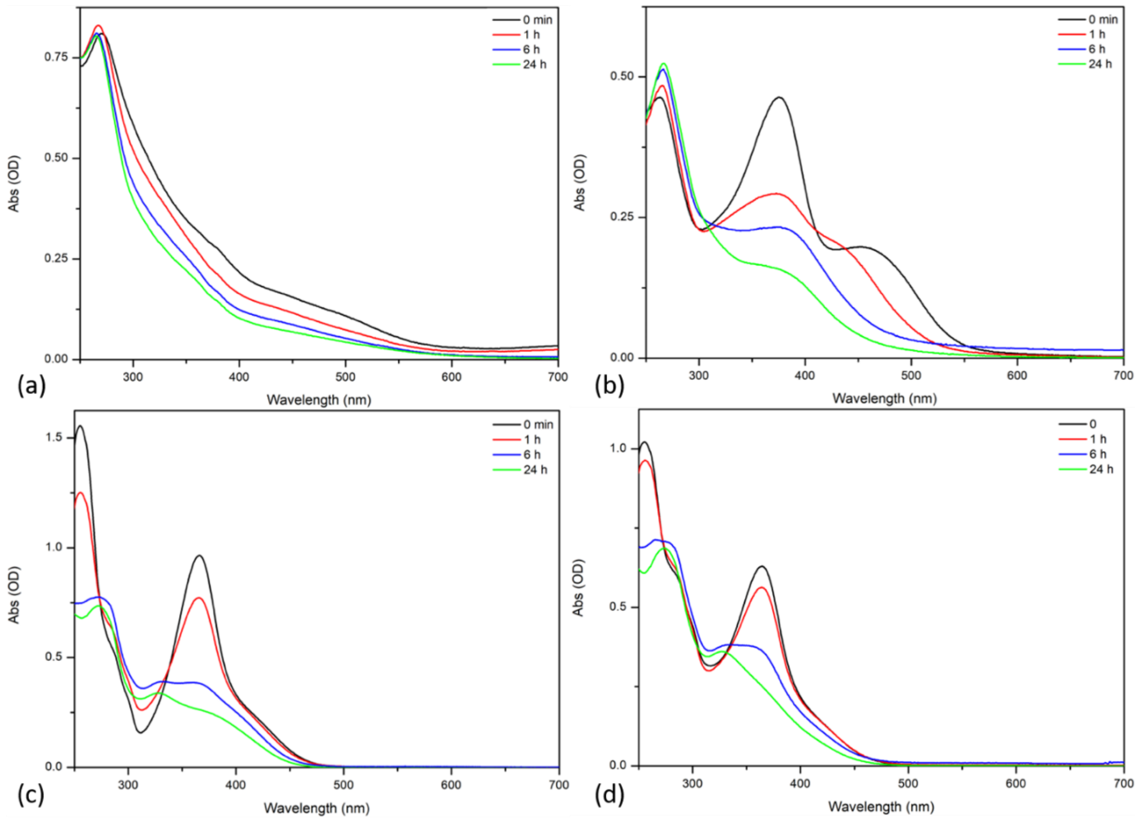

Figure S4. Stability of (a) RA, (b) CA, (c) BE and (d) BA at pH 12.

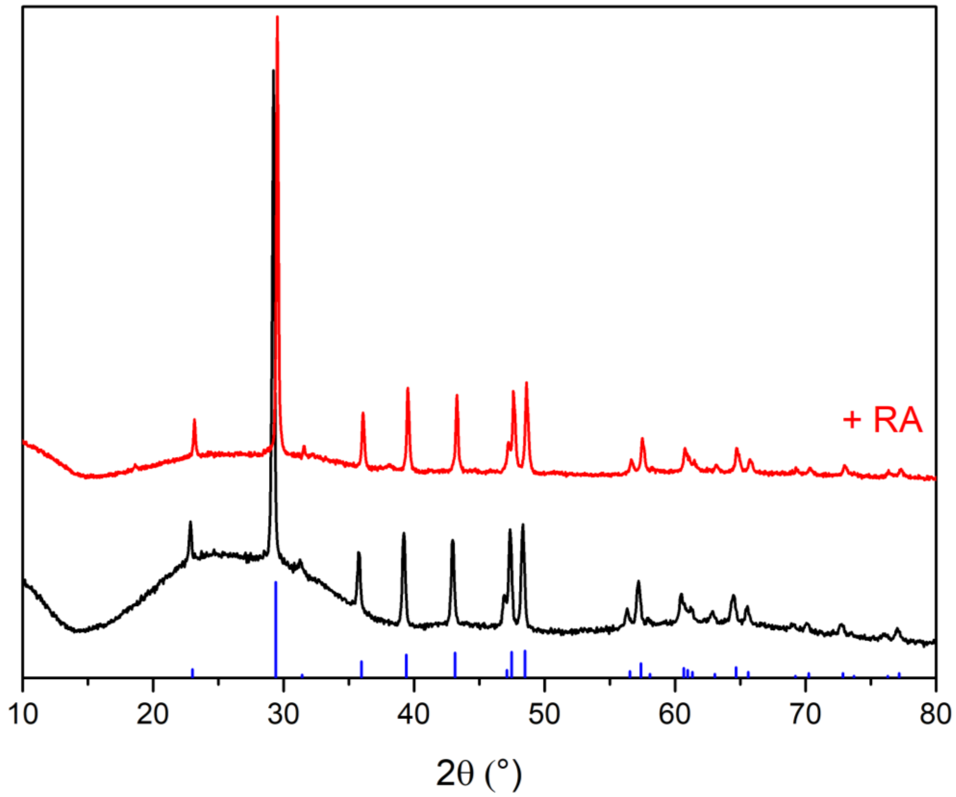

Figure S5. XRD of powder recovered after 30 min in solution of $Ca(OH)_2$ and $Ca(OH)_2$ + RA, and comparison with XRD of calcite (PDF card 05-0586).

Table S1. Synthesis of HAp NPs in presence of polyphenols at 0.05 mM and 0.5 mM: yield, particles dimensions as measured from TEM, crystallite size calculated from XRD data, incorporated quantity measured from TGA and estimated final polyphenol: Ca molar ratio.

Sample	Initial concentration of polyphenol (mM)	Yield (%)	Particle length/width (nm)	Crystallite size (nm)	Incorporated quantity ($mg.g^{-1}$)	Final molar ratio polyphenol : Ca
**HAp**	-	66	30±7/8±4	15±3	-	-
**HAp-RA**	0.05	46	14±2/4.2±0.5	16±1	52	0.015
	0.5	-	-	-	-	-
**HAp-CA**	0.05	56	19±2/6±2	15±1	42	0.011
	0.5	43	-	14±2	73	0.02
**HAp-BA**	0.05	46	13±2/4.2±0.5	13±2	54	0.012
	0.5	50	-	18±2	111	0.025
**HAp-BE**	0.05	53	17±3/7±1	17±1	52	0.02
	0.5	42	-	14±3	135	0.05
